# Spatiotemporal pH Heterogeneity as a Promoter of Cancer Progression and Therapeutic Resistance

**DOI:** 10.3390/cancers11071026

**Published:** 2019-07-20

**Authors:** David E. Korenchan, Robert R. Flavell

**Affiliations:** 1 Department of Radiology and Biomedical Imaging, University of California, San Francisco, CA 94143, USA; 2 Department of Pharmaceutical Chemistry, University of California, San Francisco, CA 94143, USA

**Keywords:** tumor microenvironment, interstitial pH, acidosis, tumor heterogeneity, magnetic resonance imaging, hyperpolarized ^13^C MRI, carbonic anhydrase, lactic acid, positron emission tomography

## Abstract

Dysregulation of pH in solid tumors is a hallmark of cancer. In recent years, the role of altered pH heterogeneity in space, between benign and aggressive tissues, between individual cancer cells, and between subcellular compartments, has been steadily elucidated. Changes in temporal pH-related processes on both fast and slow time scales, including altered kinetics of bicarbonate-CO_2_ exchange and its effects on pH buffering and gradual, progressive changes driven by changes in metabolism, are further implicated in phenotypic changes observed in cancers. These discoveries have been driven by advances in imaging technologies. This review provides an overview of intra- and extracellular pH alterations in time and space reflected in cancer cells, as well as the available technology to study pH spatiotemporal heterogeneity.

## 1. Introduction

In their seminal paper on the hallmarks of cancer [1], Hanahan and Weinberg proposed several common features of neoplasia, largely caused by genomic changes, that promote tumor development. Although genetic mutation is a necessary component of tumorigenesis, this must necessarily be accompanied by disruptions in cellular homeostasis, reflected in changes in metabolism and transport, which can be adapted according to cellular needs. These modifications both craft what has been referred to as the 'tumor microenvironment”, an extracellular milieu that further promotes tumor development and inhibits antitumor immune activity, as well as cell–cell heterogeneity within tumors, which a growing body of research is supporting as a crucial factor in understanding overall tumor function [2]. 

Alterations in pH in cancer represent one of the principal known disruptions in cellular and tissue homeostasis. While initial interest was sparked by the observation that tumor tissues are significantly more acidic than their normal counterparts, recent research has delved into how intracellular and extracellular pH changes play a role in promoting tumor initiation, growth, survival, and metastasis. The body of research on pH suggests that global measurements of pH do not capture the full story; rather, the ability of cells to tune pH locally between organelles or between cells, as well as to respond to kinetic changes affecting pH, plays a crucial role in the development and maintenance of the cancer phenotype. 

In this review article, we will summarize known findings on pH alterations in cancer and suggest how spatiotemporal heterogeneity in pH works to promote tumor survival and progression. We will also discuss the available methodologies for measuring pH on spatial and temporal scales, as well as potential opportunities for further technical development in elucidating how pH influences tumor behavior.

## 2. pH Heterogeneity in Space

**Figure 1:**
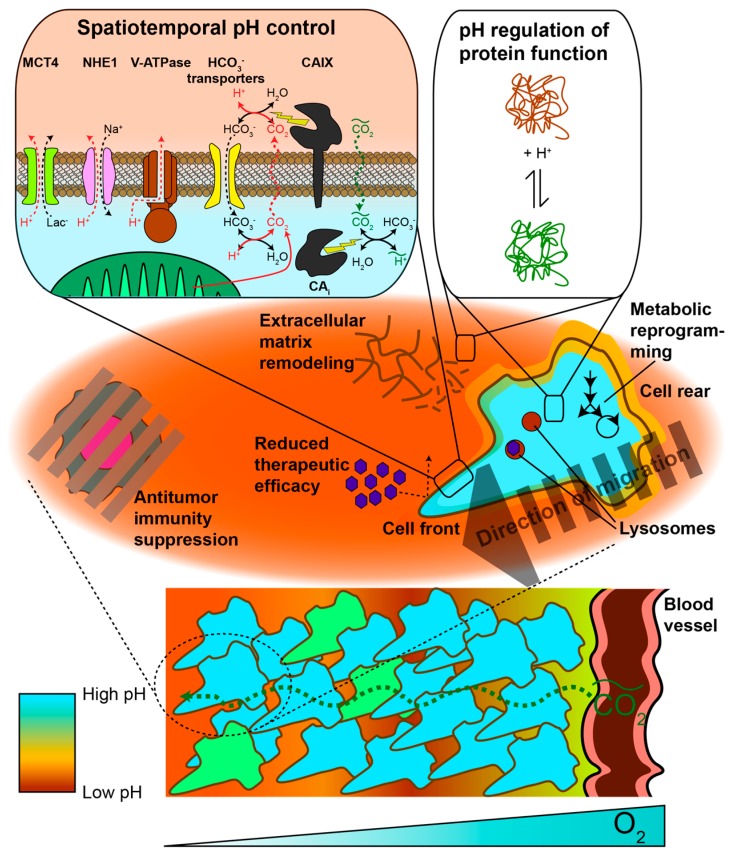
Spatiotemporal pH heterogeneity in cancer. Upper right inset: Proton extrusion mechanisms (red arrows and labels) employed by tumor cells include transport proteins such as monocarboxylate transporter 4 (MCT4), sodium-proton exchanger 1 (NHE1), or vacuolar-type ATPase. Alternatively, protons can be titrated with imported bicarbonate (HCO_3_-), which then diffuses out of the cell as CO_2_. Carbonic anhydrase 9 (CAIX) and potentially other extracellular isoforms catalyze bicarbonate-CO_2_ exchange in order to reduce CO_2_ back-diffusion into cells and induce interstitial proton release. High proton extrusion flux leads to an acidic pH_e_. Cells may also experience systemic fluctuations in CO_2_ (green arrows and labels), which induces pH_i_ fluctuations in cells expressing intracellular carbonic anhydrase isoforms. Upper left inset: Variations in pH lead to alterations in protonation states of proteins with pH-sensitive amino acid residues, thereby causing structural changes that affect protein function. Middle inset: Cancer cells may alter pH on a subcellular basis. Intracellular and extracellular pH spatial heterogeneity can promote focal adhesion formation and/or degradation for cellular migration. Additionally, altered lysosomal pH can facilitate drug resistance. An acidic pH_e_ is associated with immune cell anergy, drug localization to the extracellular space, and extracellular matrix remodeling. Lower section: pH heterogeneity may also exist on the level of tissues. Certain tumor cells may lower their pH_i_ in order to reduce proliferation and maintain capacity for differentiation. pH_e_ gradients can be sculpted in a tumor depending on metabolic differences between cells (e.g., glycolytic vs. oxidative metabolism) in combination with the proton extrusion mechanisms employed. Finally, systemic CO_2_ fluctuations can alter pH depending on CA expression and localization.

Interstitial acidification in cancer has been known for several decades, based upon electrode pH measurements [3]. More recently, it has been suggested that interstitial acidification may be accompanied by cytosolic alkalinization. There is also growing interest in the altered pH of subcellular compartments, including endosomes and lysosomes. Several excellent reviews have summarized properties and associated phenotypic changes of both intra- and extracellular pH in the context of cancer [4,5,6]. Some salient details will be mentioned here. Normal tissue may demonstrate an intracellular pH (pH_i_) and an extracellular pH (pH_e_) of about 7.2 and 7.4, respectively. In cancer, pH_e_ decreases to 7.0 or lower, leading to a reversal in the pH_i_–pH_e_ gradient across the cell membrane. Somewhat more controversial is the claim that pH_i_ significantly increases in the context of cancer, although in silico models based upon enzymatic pH-dependent activity profiles suggest that an alkaline pH_i_ confers maximal cell proliferation, upregulated glycolysis, and survival under hypoxia [7]. Changes in pH are closely linked with alterations in membrane transporter expression and function, which change transport kinetics of protons and small ions including sodium, chloride, bicarbonate, and lactate [8]. As will be discussed, pH_i,e_ changes can lead to altered protein behavior and are associated with many phenotypic changes in cancer progression, including invasion, proliferation, stemness, aggressiveness, immune suppression, vascularization, and metastasis. Figure 1 summarizes pH-related changes in transporter expression, protein function, and cellular phenotype that are associated with cancer.

### 2.1. Protonation as a Post-Translational Modification

Modulation of protein function based on pH represents a regulatory mechanism that can be rapid as well as locally constrained. Small changes in local pH can significantly affect the ratio of protonated versus deprotonated amino acid residues on a protein, depending on the acid dissociation constant (pK_a_) of the side chain. Thus, single amino acid mutations in which the side chain pK_a_ moves in or out of the physiological pH range (e.g., arginine to histidine, or vice versa) can result in either a gain or loss of protein pH sensitivity. These pH-related changes in function are well-described for particular proteins in a recent review [9]. A few examples are detailed below. Proteins that contain pH-sensitive residues or domains affecting activity include calcineurin [10], sodium-proton exchanger 1 (NHE1) [11], cofilin [12], talin [13], and cancer signaling proteins including endothelial growth factor receptor (EGFR), and transcription factor p53 [14]. Proteins found in the extracellular space, notably proteases such as matrix metalloproteinase 3; urokinase-type plasminogen activator; and cathepsins B, D, and L, also exhibit pH-dependent activity and may in fact only be activated at low pH_e_ values [15]. Interestingly, arginine-to-histidine mutations feature prominently in a subset of cancers, including acute myeloid leukemia, colorectal, esophageal, low grade glioma, kidney chromophobe, medulloblastoma, pancreatic, prostate, stomach, and uterine malignancies, suggesting that these cancers may have accompanying changes in pH_i_ to regulate protein activity [16]. Thus, by tuning pH locally and temporally tumors can alter intracellular and extracellular protein functionality.

### 2.2. Intracellular pH

Generally speaking, interest in pH_i_ changes (including subcellular compartments other than the cytosol) has surrounded alterations in protein expression and function as a result of protonation state. We will discuss cellular changes that result from intracellular pH alterations.

#### 2.2.1. Spatial Regulation of Protein Activity via Subcellular pH Heterogeneity

Localized control of pH_i_ within subcellular compartments can be accomplished based upon expression and localization of proton and other ion transporters. Spatial heterogeneity in pH_i_ can therefore contribute to phenotypic hallmarks of cancer. During cell migration, a high pH_i_ at the cell front induces formation of focal adhesion complexes, mitigated in particular by pH-dependent talin-actin binding [13], whereas a low pH_i_ at the cell rear promotes focal adhesion destabilization as well as myosin contraction [15]. Localization of NHE1 or MCT4 at the leading edge of migration can generate the higher pH_i_ required for cellular adhesion. Endosomes and lysosomes have a markedly reduced pH compared to the cytosol (6.2 and 4.5–5.0, respectively) [17], and the degree of acidity plays a crucial role in regulating lysosomal protein function. Lysosomal pH within tumor cells may therefore provide information regarding chemotherapeutic resistance, as increased vacuolar-type ATPase (V-ATPase) expression is linked with drug localization in lysosomes [18].

#### 2.2.2. Intercellular pH_i_ Heterogeneity within Tumors

An intriguing area of investigation involves the influence of pH_i_ in cellular heterogeneity within a tumor. This is because an individual cell’s fate and function can be strongly affected by pH_i_. Studies of pH_i_ in the context of eye development in Drosophila melanogaster has revealed that increased proton efflux and a resultant rise in pH_i_ are sufficient to induce dysplasia, and that proton efflux inhibition in cancer cell lines induces lethality [19]. Additionally, extracellular ATP was shown to cause intracellular acidification in prostate cancer cells, leading to growth arrest via disruption of Ca^2+^ homeostasis [20]. pH_i_ is tightly regulated, and pharmacologic inhibition of proton export significantly reduces tumor growth [21]. These studies suggest a link between a high pH_i_ and oncogenic events and proliferation in mammalian cells. However, pH_i_ plays a role in both normal development and oncogenesis. Increases in pH_i_ were shown to be necessary for the efficient differentiation of both Drosophila follicle cells and mouse embryonic stem cells. Presumably, cancer stem cells might tend to promote a lower pH_i_ than surrounding cancer cells in order to prevent differentiation until necessary. Thus, measuring pH_i_ heterogeneity between cells in a tumor mass could potentially distinguish functional regions of the tumor for the purpose of designing therapeutic strategies to disrupt intercellular symbiosis.

### 2.3. Extracellular pH

Far from being merely a side-effect of increased metabolic fluxes, the lower pH_e_ observed in tumors can also vary spatially, forming gradients within the interstitial space as well as cooperating with the alkaline pH_i_ to generate a tumor environment favorable for therapeutic resistance and metastasis. We will briefly discuss the current mechanisms underlying interstitial acidification, then summarize the effects of pH_e_ spatial heterogeneity on tumors.

#### 2.3.1. Metabolic and Physiological Contributors to Spatial Gradients and Acidic pH_e_

Interstitial acidification, independent of its tumor-promoting properties, is generally viewed as a byproduct of altered metabolism coupled with changes in perfusion within tumors. Free protons diffuse with a diffusion constant of ~1 × 10^−4^ cm^2^/s, which is fast enough compared to proton export rates to diminish the formation of spatial gradients outside the cell. However, proton diffusion through gels simulating the extracellular matrix has been measured as ~6 × 10^−6^ cm^2^/s [22], almost two orders of magnitude slower than free proton diffusion and on the same order as water diffusion through biological tissue (~2 × 10^−6^ cm^2^/s) [23]. This suggests that protons largely diffuse as mobile buffer species such as phosphate or carbon dioxide. As a cancer grows and outdistances its local blood supply, tumor regions become hypoxic, although they remain with diffusive distance of glucose [24]. This has traditionally been understood to lead to HIF-1α stabilization, which in turn induces overexpression of glycolytic enzymatic subunits (e.g., LDHA), proton-exporting transporters such as monocarboxylate transporter 4 (MCT4), and carbonic anhydrase 9 (CAIX). Increased lactate metabolic flux coupled with proton-lactate co-export via MCT4 is generally accepted to be a major mechanism of interstitial acidification, with a variety of other mechanisms also contributing (Figure 1). For example, V-ATPase and MCT4 are both major acidification mechanisms in human breast cancer cell lines [25]. Acidification has also been shown to occur in glycolysis-deficient Chinese hamster cells [26,27], suggesting that proton export pathways independent of lactate play a major role in this particular model. Therefore, depending upon the genetic and metabolic state of the tumor, a variety of mechanisms contribute to interstitial acidification.

Recent studies have suggested that pH_e_ gradients may be present throughout a tumor mass, and that these gradients, as well as the pH_i_–pH_e_ gradient across the cell membrane, promote tumor growth and survival. Experimental evidence suggests that free proton diffusion through tumor interstitium is very small and that the majority of proton diffusion takes place by way of mobile buffers [22]. It has been hypothesized that various interstitial acidification mechanisms may be present throughout a tumor mass depending on oxygen/metabolite availability, transporter expression, and carbonic anhydrase (CA) activity, contributing to a pH gradient throughout the tumor [6]. Additionally, stromal cells near a lesion may shape pH_e_ gradients by forming a syncytium that can take up acidic byproducts and transport them away from the site of metabolism, as has been demonstrated in co-cultures of myofibroblasts and colorectal cancer cells [28]. An intriguing area of research is the contribution of CAIX to gradient sculpting of both pH_i_ and pH_e_ within cell spheroids and in vivo xenografts. HCT116 human colon carcinoma spheroids transfected to constitutively express CAIX diminish pH_i_ gradients between the spheroid core and periphery [29] while simultaneously increasing pH_e_ gradients [30]. When these cells were implanted in a mouse and imaged with a ^1^H MRSI pH agent, the resulting tumors only demonstrated voxel pH_e_ values below 6.93, suggesting that CAIX acts as a “pH-stat” and keeps pH_e_ below a certain level [31]. Another interesting finding is that acid-extruding bicarbonate transporters such as solute carrier family 4, members 4 and 9 (SLC4A4 and SLC4A9) are hypoxia-inducible and are therefore likely expressed along with CAIX in hypoxic tumor regions [32]. Mathematical modeling of bicarbonate-CO_2_ exchange, diffusion, and cellular metabolism predicts that the effect of CAIX catalysis on pH_i_ and pH_e_ heterogeneity is strongly dependent upon metabolic pathways and proton transport mechanisms. Whereas cells that mainly produce CO_2_ or import HCO_3_- to titrate intracellular protons benefit greatly from higher CAIX activity, cells which export protons directly are minimally or even negatively affected by expressing CAIX [33]. Thus, interactions between cellular metabolism, proton export, CO_2_ diffusion, and bicarbonate-CO_2_ interconversion all give rise to the pH_e_ observed in tumors. 

#### 2.3.2. Acidic pH_e_ Can Alter Tumor Metabolism

Interstitial acidification that may be caused by altered metabolism can in turn affect metabolic pathways. Ippolito et al. discovered in a prostate neuroendocrine carcinoma (PNEC) cell line that glutamate decarboxylase (GAD) activity in cell lysates increased when the cells were incubated in acidic media (pH 6.5) and decreased in alkaline media (pH 8.5) [34]. Interestingly, glutamate-ammonia ligase (GLUL) demonstrated higher activity in both acidic and alkaline media relative to physiologic pH (pH 7.4). The measured activities correlated with changes in protein expression as well. In a following study, the group discovered that altering the culture media pH significantly altered metabolic pathways in the same cell line, with acidic pH favoring oxidative phosphorylation and alkaline pH stimulating nutrient consumption [35]. In keeping with this finding, they demonstrated that PNEC and human prostate cancer cell lines were more susceptible to niclosamide inhibition of mitochondrial function at acid pH and more susceptible to nutrient deprivation at alkaline pH. These findings are intriguing in that they suggest that pH_e_ measurements can aid in identifying metabolic heterogeneity within a tumor in order to devise therapeutic strategies against tumor subregions.

#### 2.3.3. Spatial pH_e_ Gradients Promote Healthy Cell Death, Tumor Aggressiveness, and Therapeutic Resistance

Spatial regulation of tumor pH_e_ has a profound impact upon tumor aggressiveness, survival, and treatment resistance. The lower tumor pH_e_ forms a gradient with the less acidic interstitium of normal tissue, promoting a net proton flow that may in turn induce normal cell toxicity [36]. Normal cells may be unable to cope with the acid load because they are less able to remove intracellular protons as effectively as tumor cells. The resulting interstitial acidification could activate caspase activity, leading to apoptosis [37]. Interestingly, loss of p53 in cancer cells seems to protect against acidic pH_e_-dependent induction of apoptosis as well [38]. Spatial fluctuations in pH_e_ can affect cellular migration and invasion. Prior in vivo studies of breast and colon cancer cells implanted in mice have established that tumors grow preferentially along gradients of decreasing pH_e_, and that overexpression of glucose transporter 1 (GLUT1) and NHE1 transporters at the leading edge drives invasion [39]. The high transmembrane pH_i_–pH_e_ gradient can also act as a defense mechanism against weakly basic chemotherapeutic agents, since these will preferentially protonate in the interstitium, pick up a net positive charge, and be prevented from cell internalization. This likely explains why extracellular alkalinization via bicarbonate buffer therapy was shown to enhance doxorubicin (pK_a_ = 7.6) uptake in MCF-7 xenografts in vivo [40]. Several effects of acidic pH_e_ on antitumor immune cell activity have been documented. Acid pH_e_-dependent lactate import can induce anergy in human cytotoxic T-cells in vitro [41], and pH neutralization in vitro reverses anergy for human and murine infiltrating T-lymphocytes [42]. Acidosis leads to greater antigen uptake and presentation in dendritic cells (DCs) [43]; however, tumor-derived lactate could also play a role in inducing a tumor-favorable phenotype in DCs [44]. Bicarbonate therapy leads to more effective tumor cell killing by natural killer (NK) cells, suggesting a role for pH_e_ in modulating NK cell activity [45]. Finally, low pH_e_ also affects cellular differentiation status. Acid pH_e_ promotes the expression of glial stem cell markers [46] and promotes epithelial-to-mesenchymal transition in Lewis lung carcinoma cells [47].

Because transmembrane proton exporters and CA isoforms play a prominent role in cancer, several inhibitors have been designed against these targets. A good review on CA inhibitors has been written by Singh et al. [48]. Other inhibitors include amiloride derivatives against NHE1, proton pump inhibitors (PPIs) against V-ATPase, and MCT inhibitors such as 4,4′-di-isothiocyanostilbene-2,2′-disulfonate (DIDS) [49]. Additionally, a large body of literature exists on designing pH-sensitive drug delivery systems [50] or prodrugs [51] that release the drug or form the active therapeutic agent when they encounter the acidic tumor microenvironment.

## 3. pH Heterogeneity in Time

Studying alterations in pH distributions around, between, and within cells does not fully capture the underlying biological changes in cancer. Alterations in tumor pH happen on various timescales, ranging from transient, rapid fluctuations to slow, progressive changes. The fast-switching ability of pH-sensing protons potentially lends itself to respond to rapid changes in pH. Furthermore, tumor cells are known to undergo hypoxic-normoxic cycles as they outdistance their blood supply [24]. These cycles likely imply associated changes in pH, which can modulate cell behavior. Additionally, changes in regulation of carbonic anhydrase isoforms can affect the timescale of transient pH changes and thus increase or decrease tumor cell sensitivity to pH transients. The study of pH modulation kinetics represents an intriguing area of tumor biology that is only beginning to be elucidated. Finally, pH_i,e_ in solid tumors can change over the course of disease progression in order to suit the changing needs and objectives of tumor cells. The following section highlights salient findings regarding temporal pH changes. 

### 3.1. Carbonic Anhydrase Kinetics

The changes in protein expression for various isoforms of carbonic anhydrase, notably CAIX, are well-known for many cancers [52]. As alluded to in Section 2.2.1, CAIX activity can enable higher metabolic fluxes for rapidly-dividing cancer cells by accelerating HCO_3_-/CO_2_-mediated “acid venting”, which clears away acidic byproducts that might otherwise back up actively-utilized metabolic pathways. Biological studies and mathematical modeling of lactic acid-producing muscle tissue demonstrate the role of extracellular carbonic anhydrase in sustaining high metabolic flux and clearance to blood [53,54]. One of the important features of CAIX in particular is that because its expression is regulated via HIF1α, it is expressed along with other metabolism-related proteins such as LDHA, MCT4, and SLC4A4/9. Additionally, although CAIX and other hypoxia-induced proteins can be expressed through non-canonical HIF pathways, the catalytic activity of CAIX may be enhanced under hypoxic conditions [55]. As mentioned in Section 2.2.1, CAIX catalysis enhances metabolic fluxes depending upon the metabolic pathways utilized by cancer cells. Therefore, measuring bicarbonate-CO_2_ exchange kinetics in vivo could serve as an indicator of metabolic capacity. Gallagher et al. measured differences in CAIX activity of HCT116 cells with and without constitutive CAIX expression implanted subcutaneously in mice. Although the CAIX-expressing cells demonstrated a faster rate of conversion in vitro, a slower interconversion was observed for these same cells in vivo, which they attributed to the lower in vivo tumor pH_e_ reducing overall CAIX enzymatic activity [56]. This highlights the complex phenomena that may contribute to pH regulation in vivo.

### 3.2. Effects of pH_i_ Transients on Tumor Cells

A very intriguing study was recently performed by Hulikova et al. regarding CA isoforms and coupling with CO_2_ fluctuations [57]. The authors discovered that although expression of intracellular CA isoforms in various cell lines did not enhance proton diffusion throughout the cytosol, they did sensitize cytosolic pH_i_ to CO_2_ fluctuations, enabling it to oscillate in response to oscillating pCO_2_. Mathematical modeling of pCO_2_-pH_i_ coupling revealed that the downregulation of intracellular CA_i_ acted as a sort of low-pass filter, reducing the amplitude of more rapid pH_i_ fluctuations. Intriguingly, the mammalian target of rapamycin complex 1 (mTORC1) pathway activation as measured by lower ribosomal protein S6 kinase (S6K) phosphorylation was achievable by exposing HCT116 cells to sharp pCO_2_ fluctuations, whereas phosphorylation state did not significantly correlate with the average pH_i_. CA inhibition with acetazolamide or knockdown of intracellular carbonic anhydrase 2 (CAII) significantly altered S6K phosphorylation state. The authors concluded that this coupling between pCO_2_ and pH_i_ represents a potent signaling mechanism that can alter cellular activity, particularly for intracellular CA-expressing tumor cells experiencing rapid pCO_2_ fluctuations. The authors also suggested that downregulation of CA_i_ isoforms may confer a survival advantage of tumor cells over healthy cells, allowing them greater control over pH_i_ as pCO_2_ fluctuates. At the same time, they proposed that cancers cells with high CA_i_ activity near aberrant blood vessels experiencing sharp pCO_2_ fluctuations would experience mTOR-dependent changes in metabolism and perfusion, elicited by changes in intracellular [Ca^2+^]. These results suggest that a technique that can measure temporal pH_i_ fluctuations could identify tumors or tumor regions that are activating particular oncogenic pathways, thereby facilitating tumor characterization. It also proposes a mechanism by which pH-sensing proteins could become activated or deactivated by temporal pH changes. 

### 3.3. Tumor pH_e_ Decreases Over Time During Tumorigenesis and Disease Progression

While rapid fluctuations in tumor pH_i,e_ may regulate cancer cell function and metabolism, pH_i,e_ changes over a slower time scale (e.g., weeks to months) may play an important role in cancer progression. For example, it has been demonstrated that changes in pancreatic pH_e_ result from chronic inflammatory pancreatitis and after secretin administration, and it has been hypothesized that this could drive pancreatic cancer development [58]. This hypothesis is further supported by evidence that low pH_e_ is associated with local invasion [39] and metastatic disease [59]. Moreover, alterations in metabolic phenotype, including increased production of lactic acid, are upregulated in disease progression, thereby inducing a higher degree of acidosis (Figure 1). These indirect lines of evidence suggest a temporal link between changes in tumor pH and an invasive, metastatic phenotype.

The hypothesis that tumor pH_e_ decreases during disease progression could be directly tested in spontaneous genetically engineered models (GEM), which allow monitoring of tumors from early precursors to high-grade, metastatic, lethal tumors [60]. Recently, we have evaluated the pH_e_ of the Transgenic Adenocarcinoma of the Mouse Prostate (TRAMP) model in both early- and late-stage tumors. This is a spontaneous GEM which proceeds from hyperplasia to low-grade tumors, ultimately progressing to high-grade disease, metastasis, and death [61]. We hypothesized that pH_e_ would decrease based on prior data indicating increased production of lactic acid in high-grade tumors [62], as well as the observation that treatment with sodium bicarbonate blocked tumorigenesis in this model [63]. Supporting the hypothesis, we found that there was a significant decrease in pH_e_ in mice bearing high-grade tumors compared against low-grade counterparts [64]. Therefore, there is an accumulating body of both indirect and direct evidence supporting the hypothesis that a temporal decrease in pH_e_ could represent a biomarker of tumor disease progression.

## 4. Techniques to Measure pH Spatiotemporal Heterogeneity

**Figure 2:**
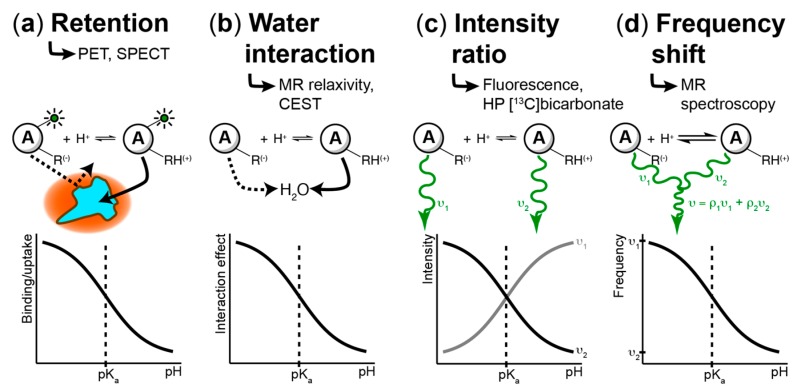
Mechanisms of pH measurement in cells and tissues. As a general rule, a pH-sensing agent must contain at least one functional group with a pK_a_ within the physiological range of detection to generate image contrast. (**a**) Agents may demonstrate pH-dependent cell binding or uptake. In this case, a pH decrease can trigger a change in cell permeability, membrane binding, or release of a prodrug agent which can bind to cells. Importantly, absolute pH quantification is not possible. This approach is used primarily for pH imaging with positron emission tomography (PET) or single-photon emission computerized tomography (SPECT). (**b**) MR-based agents may interact with water protons in a pH-dependent manner, in which pH induces changes in relaxivity or in exchange (as in acido-chemical exchange saturation transfer, acidoCEST). (**c**) The protonated and deprotonated states of an agent may emit different electromagnetic frequency waves. In this case, the ratio of emission between the two wavelengths can be used to determine the pH. This approach is relevant to fluorescent-based pH probes as well as hyperpolarized (HP) [^13^C]bicarbonate. (**d**) If the kinetic rate of protonation–deprotonation is much faster than the absolute frequency difference between emission wavelengths, the agent will exhibit a frequency shift rather than two distinct emission wavelengths. The observed frequency depends on the relative populations (ρ) of protonated and deprotonated states, thereby giving the pH. This approach describes pH imaging with MR spectroscopic techniques (^1^H, ^31^P, HP ^13^C).

The interstitial acidification that accompanies cancer was first discovered using pH microelectrodes [3]. Today, many methodologies exist for studying pH changes in time and space, covering a wide range of modalities, including nuclear methods such as positron emission tomography (PET) and single-photon emission computerized tomography (SPECT), fluorescence, and magnetic resonance. The principles underlying chemical agents that detect pH changes are summarized in Figure 2. These techniques rely on a variety of mechanisms to generate image contrast, and they operate over a wide range of spatial resolution scales. To the best of our knowledge, the only techniques that have reported tumor pH_e_ in patients are microelectrodes (5.85–7.68 over many tumor types) [3], ^11^C-DMO PET (6.88–7.26 in brain tumors/metastases) [65], and acido-chemical exchange saturation transfer (acidoCEST) MRI (6.58 in metastatic ovarian cancer) [66]. In this section, we will briefly summarize the range of techniques available for studying pH heterogeneity; these have also been discussed in a recent review [67].

### 4.1. Fluorescence-Based Measurements

Various fluorescent dyes and proteins exhibit a wavelength shift upon protonation, enabling ratiometric pH_i_,_e_ calculation by measuring fluorescent output at each wavelength. In general, fluorescence methods including microscopy have very high spatial resolution (<1 µm), allowing subcellular measurements of pH gradients, although applications to whole animal and clinical imaging are limited by low penetration of light through tissue. An exemplary review covering pH-sensitive fluorescent dyes is Han et al. [68]. Generally, pH imaging studies with fluorescent dyes are constrained to 60 minutes in length. One notable approach to pH measurement in various intracellular compartments involves transfecting cells with a genetic construct encoding a pH-sensitive fluorescent protein (e.g., pHluorin) modified with a targeting domain that will localize the protein to the desired organelle [69,70]. This approach also extends the imaging timescale beyond that achievable with fluorescent dyes. The low depth of tissue penetration for fluorescence in the visible spectrum can be overcome through the use of agents emitting in the near-infrared range, which have a deeper tissue penetration compared with visible light [71,72], allowing imaging in murine models. A different way to overcome the tissue penetration limitations of fluorescence dyes in preclinical in vivo studies is by constructing a dorsal window chamber. Some studies have demonstrated the use of SNARF-1 fluorescent dye with a dorsal window chamber in order to study proton gradients and flow in tumor tissue [36] and to observe tumor cell migration along pH_e_ gradients [39]. In theory, a similar approach could be utilized for intraoperative fluorescence imaging of pH in patients, which has been reported using other fluorescence molecular imaging probes [73], although this has not been reported to date.

### 4.2. PET/SPECT-Based Imaging Methods

Several radiolabeled tracers have been developed in order to study pH_e_ in vivo, including ^11^C-dimethyloxazolidinedione (DMO) [65], ^11^CO_2_ [74], and ^123^I-labeled derivatives of malonic acid [75]. One elegant example of probe design is ^64^Cu-conjugated pH-low insertion peptide (pHLIP), which anchors the radioisotope into cell membranes in regions where pH_e_ is below 7.0. We have reported the synthesis of various caged derivatives of ^18^F-fluorodeoxyglucose (FDG) that demonstrate pH_e_-sensitive localization in vivo by caging group release in acidic pH_e_ followed by uptake via glucose transporters. By using different amine-containing caging groups varying in pK_a_, we could tune the pH sensitivity of FDG uptake [76]. PET/SPECT-based approaches can be readily implemented for in vivo imaging with good spatial resolution (1–2 mm); however, their primary limitation is that they cannot measure absolute pH_e_, but only indicate regions below a certain threshold pH_e_. Although arterial blood sampling along with imaging can be fit to a model to estimate pH values [65,74], these pH values have not been correlated with microelectrode measurements. Nevertheless, these techniques may prove to be useful in a clinical setting if threshold pH values can be demonstrated to sensitively and selectively identify or characterize lesions.

### 4.3. MR-Based Techniques

Magnetic resonance offers spectral sensitivity that enables absolute pH quantification with high tissue penetration depth, making it a well-studied technique for pH_i,e_ measurement. Major techniques under investigation include spectroscopic methods and chemical exchange saturation transfer (CEST) based methodologies. 

#### 4.3.1. Chemical Exchange Saturation Transfer (CEST)

CEST approaches represent a rapidly-developing field of study for pH_e_ measurement which has the advantage of high spatial resolution (0.1–2 mm) but potentially low sensitivity; a comprehensive review has been written by Chen et al. [77]. Generally, CEST techniques are able to measure pH_e_ by determining relative rates of protonation and deprotonation, typically for amide functional groups on either endogenous molecules or administered contrast agents. A recent study demonstrated more robust and accurate measurement of pH_e_ using the acidoCEST method, which relies upon exogenous contrast agent administration [78]. In an elegant study, Longo et al. combined CEST pH_e_ measurements with FDG PET imaging, demonstrating an inverse correlation between tumor pH_e_ and glucose uptake [79]. It is noteworthy that acidoCEST with the FDA-approved CT contrast agent iopamidol to measure pH_e_ has been demonstrated in patients with high-grade invasive ductal carcinoma and with metastatic ovarian cancer [66]. 

#### 4.3.2. MR Relaxometry

In this method, paramagnetic contrast agents with a predictable, pH-dependent change in spin-lattice (T_1_) relaxation time are administered and used to measure tissue pH. A major strength of this method is high signal-to-noise ratio and spatial resolution (0.2–2 mm); however, a second, pH-insensitive contrast agent must also be administered to accurately measure pH. For example, Gillies et al. used a pH-dependant chelate, GdDOTA-4AmP^5−^, paired with a pH-independent analog, GdDOTP^5−^, to generate high spatial resolution maps of tissue pH_e_ in a rat glioma model [80]. A variety of other agents have been reported [81,82] for this purpose. 

#### 4.3.3. MR Spectroscopic Approaches

Magnetic resonance spectroscopy can be used to measure tumor pH_i,e_ by measuring the chemical shift of a nucleus in a molecule with a pK_a_ close to the physiological range and determining the pH from a previously-constructed MR titration curve. This has been described for a variety of chemical compounds and nuclei, including ^31^P, ^1^H, and ^19^F. ^31^P MR spectroscopy can be used to measure pH_i_ based on the chemical shift of the inorganic phosphate peak. Administration of 3-aminopropylphosphonate (3-APP) followed by ^31^P MR spectroscopy can be used to measure pH_e_ [83,84,85]. Similarly, ^1^H MR spectroscopic imaging of imidazole-containing compounds, notably (±)2-(imidazol-1-yl)3-ethoxycarbonylpropionic acid (IEPA) [86] and (±)2-(imidazol-1-yl)succinic acid (ISUCA) [31,87,88], can generate in vivo pH_e_ maps and therefore be used to study pH_e_ heterogeneity. Similar approaches have been reported with ^19^F-containing agents [89]. With the exception of ^31^P, MR imaging approaches can capture pH_e_ heterogeneity with acceptable spatial resolution (1–2 mm) and in a reasonable timeframe (10–30 min), however it cannot measure pH_i_ or capture kinetic pH changes. Both these limitations are linked with low signal-to-noise or contrast-to-noise ratios.

#### 4.3.4. Hyperpolarized (HP) ^13^C MR Imaging

**Figure 3:**
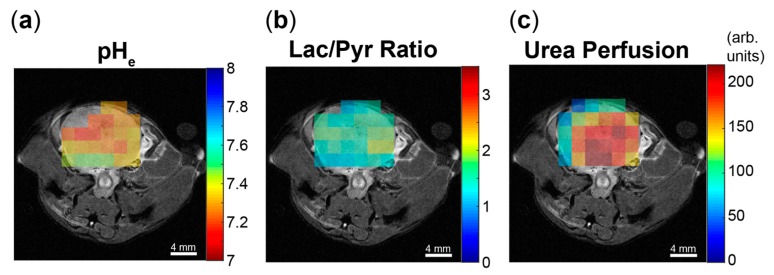
Hyperpolarized imaging of pH_e_, lactate conversion, and perfusion can be performed in a single imaging study. Data are shown for a transgenic adenocarcinoma of the mouse prostate (TRAMP) animal model displaying a consolidated, high-grade tumor confirmed with histology. HP images of (**a**) extracellular pH, (**b**) lactate-to-pyruvate (Lac/Pyr) ratio, and (**c**) urea signal intensity are shown overlaid on ^1^H anatomical images, enabling voxel-to-voxel correlations in order to study the interplay between metabolism and acidification.

The dramatic gain in MR signal attainable through dissolution dynamic nuclear polarization (d-DNP) [90] provides unique opportunities to capture spatial as well as temporal changes in pH_e_. Importantly, the ability to polarize, inject, and image multiple agents simultaneously [91] or in the same imaging session holds great promise for simultaneously measuring pH and related metabolic or physiological processes, such as glycolysis and perfusion. pH_e_ mapping with HP [^13^C]bicarbonate represents the majority of HP pH imaging and has been demonstrated in tumors [91,92,93], perfused lungs [94,95], and other tissues [96,97]. pH_i_ can also be quantified from HP ^13^CO_2_ produced from [1-^13^C]pyruvate in organs with high pyruvate dehydrogenase flux, most notably the heart [98,99]. Although the short T_1_ of [^13^C]bicarbonate/^13^CO_2_ (~10 s in vivo [91,92,93,97,100]) poses a significant challenge for obtaining sufficient spatial resolution, recent advances in hyperpolarization approaches, including HP precursor decarboxylation [93,94,101], as well as advanced HP imaging sequences [96,102,103], can provide significant gains in available HP signal and its effective utilization. Nevertheless, HP image resolution (2–10 mm) is currently coarser than other modalities and MR approaches. Figure 3 demonstrates hyperpolarized pH_e_ imaging using an optimized [^13^C]bicarbonate method along with HP measures of glycolysis and perfusion in the TRAMP mouse model of prostate cancer. Other ^13^C-labeled compounds have also demonstrated pH sensitivity that are potentially amenable to in vivo pH_e_ imaging, including N-(2-acetamido)-2-aminoethanesulfonic acid (ACES) [104], diethylmalonic acid (DEMA) [105], zymonic acid [106,107], and amino acid derivatives [108]. Notably, zymonic acid was applied to pH imaging in kidneys and in a mammary tumor model [106]. Taken together, these data demonstrate robust tumor pH_e_ measurements using a variety of HP ^13^C MRI methods.

Importantly, high HP signal gains can also be used to measure kinetic phenomena, such as CA-catalyzed [^13^C]bicarbonate-^13^CO_2_ exchange, on a sufficiently short timescale (0.1–1 s resolution). Gallagher et al. studied differences in both pH_e_ and bicarbonate-CO_2_ exchange within HCT116 xenografts that either overexpressed CAIX or did not [56]. The bicarbonate-to-CO_2_ forward reaction rate was quantified by selective saturation of the HP ^13^CO_2_ resonance. They found that CAIX-overexpressing tumors demonstrated a pH_e_ that was 0.15 units lower, similar to results in cell spheroids [30], but a paradoxically slower exchange rate, which they attributed to a pH-dependent reduction in CAIX activity. It may be possible to measure both pH_e_ and exchange rate in vivo through MR spectroscopic techniques similar to the ones employed in this study, although the injected HP [^13^C]bicarbonate must be given sufficient time to equilibrate if pH_e_ is to be accurately measured.

HP approaches may also enable in vivo kinetic measurements of pH_i_, which represents a tantalizing subject of investigation. One elegant example of this was demonstrated in perfused rat hearts, where hyperpolarized [1-^13^C]pyruvate was decarboxylated to form ^13^CO_2_, enabling intracellular measurement of pH by comparison with the bicarbonate resonance. The HP bicarbonate/CO_2_ data clearly show differences in measured pH_i_ dynamics with and without CA inhibition, suggesting the ability to quantify CA-catalyzed intracellular bicarbonate-CO_2_ interconversion [98]. Similarly, HP ^13^C-labeled organic phosphates formed from [U-^13^C,U-^2^H]glucose, including glyceronephosphate and 3-phosphoglycerate, have been shown to enable pH_i_ quantification in yeast cells [109]. A similar approach in which a HP ^13^C-labeled compound is taken up in mammalian cells and subsequently phosphorylated to generate a pH-sensing moiety that may be feasible. Additionally, an existing HP pH agent such as a dicarboxylic acid could be derivatized to form an ethyl ester, as has been demonstrated with [1-^13^C]pyruvate to enhance blood–brain barrier crossing [110]. The ester groups could then be cleaved inside the cell to generate the carboxylic acid moieties and regain pH-sensing ability. Both these approaches may introduce significant toxicity concerns; nevertheless, these or other HP approaches could open the way to measuring pH_i_ spatiotemporal fluctuations in vivo. 

## 5. Conclusions

Measuring average pH in tumors provides useful information but fails to describe the complex dynamics of the tumor microenvironment. The spatial heterogeneity surrounding, among and within cells plays a major role in driving the aggressive tumor phenotype. Rather than serving as merely a byproduct of altered metabolism, pH variation throughout a tumor generates the necessary conditions to alter protein functionality throughout cells, preserve cellular differentiation capacity, reduce therapeutic uptake, shut down antitumor immune activity, and promote cellular migration and metastasis. In addition, the kinetic processes that influence pH may hold a great deal of information regarding tumor initiation, metabolism, and cellular maintenance. Many imaging and measurement techniques have been developed in order to study both pH_i_ and pH_e_. Hyperpolarized ^13^C in particular holds great promise for capturing both spatial and temporal heterogeneity within tumors based on its ability to estimate kinetic rate constants in an imaging setting. Further development of these and other in vivo pH measurement techniques will help to reveal the complex role that proton transport plays in tumor development and therapy.
